# “Don’t Stop the Music,” Please: The Relationship between Music Use at Work, Satisfaction, and Performance

**DOI:** 10.3390/bs13010015

**Published:** 2022-12-24

**Authors:** Domenico Sanseverino, Andrea Caputo, Claudio Giovanni Cortese, Chiara Ghislieri

**Affiliations:** Department of Psychology, University of Turin, 10124 Turin, TO, Italy

**Keywords:** music, job satisfaction, performance, structural equation model

## Abstract

Although there are several studies in the literature that have examined how different types of music affect performance or other organisational outcomes, knowledge about how music affects individuals in the workplace is still limited, especially in terms of perceived music use. This study aims to examine the effects of three different uses of music—namely, emotional, cognitive, and background music—on individual perceptions of job satisfaction and performance. A sample of 244 workers from different backgrounds was included in the study. We tested a full structural equation model. The results show that (1) emotional use has a positive relationship to performance, both directly and indirectly through mediating job satisfaction; (2) cognitive use has no significant effect on satisfaction and performance (even indirectly); and (3) background use has a negative relationship to job satisfaction and no relationship to performance. This work suggests that listening to music during work activities could be a positive organisational practice. Future studies could further investigate the role of music listening as a resource, taking into account other personal and contextual characteristics.

## 1. Introduction

Research on the effects of listening to music at work is relatively underdeveloped, though its roots can be traced back to the beginning of the last century, especially in relation to industrial music [1,2]. The industrial perspective viewed music as a “fair” motivational tool to enhance performance. To cite one of the most famous examples, the BBC created a radio program called *Music While You Work*, which was broadcast daily in British factories from 1940 to 1967 to increase worker productivity [3]. Although performance was the focus of these early studies, the results were often inconclusive: Uhrbrock [4] pointed out in his review that music was likely to produce only a temporary increase in performance in inexperienced workers who had simpler tasks to perform. On the other hand, Fox [5] argued that music was a means of maintaining workers’ attention in the face of the inevitable lapse in alertness caused by repetitive work; that is, music does not increase productivity per se, but instead maintains baseline levels. It can be argued that such a view of work performance falls short, given that even the earliest studies found that music boosted morale and that workers were generally opposed to removing music once it was introduced [6]. Workers may not have actually worked harder, faster, or better, but they felt better, and as is generally recognized in the literature, subjective well-being is a protective factor against negative workplace outcomes [7].

Today, much of work has shifted from factories to offices and from repetitive, simple tasks to more complex, cognitive tasks that often include customer service. In addition, technological advances—especially in the last twenty years—have led to a shift in the accessibility of music. This development is not new. In the first decade of the 21st century, it was already noted that music can no longer be considered a commodity, but was instead an easily accessible resource [8]. In recent years, the accessibility and consumption of music has even increased, especially during the COVID-19 pandemic.

Today, autonomy in choosing and controlling music during work activities is unprecedented: people are freer to choose when they listen to music, what genre they listen to, and, perhaps more importantly, they have a greater degree of control over the function they believe music has for them. More generally, mood and performance can be influenced by the various experiences that occur when listening to music. This influence may be the direct result of an emotional experience or an aesthetic perception, i.e., an understanding of the musical elements [9]. This already poses an additional challenge when attempting to study the effects of music, as the intention behind the choice of listening may alter the desired outcomes. As Thorsén [10,11,12] found, workers in Volvo factories wanted to listen to music primarily for two reasons: to focus more on work tasks and to escape from work. This suggests that music has different functions at work and its effect should be considered to be context-dependent [13], i.e., music can serve multiple functions in different situations, such as promoting concentration or during a break. Moreover, music use is also influenced by personality [14].

Given the sudden increase in remote work, now followed by a gradual return to in-person work, and the relative lack of research in recent years on workers’ subjective experiences of music in the workplace, this study provides a preliminary examination of the effects of differential use of music on perceived organisational outcomes.

### 1.1. Music and Job Performance

There are few studies that have examined the effects of music on work performance, and they have controversial results. In this context, we focus on task performance, i.e., activities that are directly related to the creation of a product or service or to the functions required to perform these activities, such that both the performance of one’s work role and the effectiveness of the organisation depend on task performance [15]. Shi, Huang, and Chiang [16] found that background music with lyrics has a negative effect on concentration and attention, while Haake [13] found that music can also support more complex cognitive and creative processes, although these can be affected by excessive levels of arousal from music. On this topic, Huang and Shi [17] found that background music negatively affected attentional performance when listeners strongly liked or disliked the music. Padmasiri and Dhammika [18] focused on workers in industry and found that listening to relaxing music lowered performance. A possible explanation for these competing results can be found by extending activation theory [19].

Music can increase worker activation, which leads to increased performance. However, this relationship is not linear, and it has been found to be moderated not only by other variables but also to depend on the task. If more cognitive resources cannot be allocated to the task, a further increase in resources for cognitive processes will not lead to an increase in performance [6]. On the other hand, music does not usually produce excessive levels of arousal [20]. It can be argued that this is especially true for music played during work, so the relationship between listening to music and activation can be considered to be linear. This also explains the opposite effect: relaxing music lowers activation, so listening to music during work is counterproductive. However, being able to freely choose preferred music, rather than having to take a test or be exposed to imposed music, may have a different effect. Lesiuk found that the positive affect and quality of work is higher when listening to music [21]. In addition, the author found that people who could listen to their preferred music whenever they wanted reported lower stress and improved mood, as well as better cognitive performance scores in situations with high cognitive demands [22].

Given these findings, it appears that there are several factors to consider when examining the effects of music on performance, but the degree of autonomy over when and how music is listened to may play an important role in whether music can have positive organisational effects.

### 1.2. Music, Positive Affect, and Job Satisfaction

Job satisfaction is a well-studied variable in organisational research and is considered an important indicator of well-being. While there are a variety of definitions in the literature, job satisfaction can be summarized as a collection of positive or negative feelings and beliefs about one’s current job that depend on a worker’s assessment of the extent to which their work experiences match expectations, especially in terms of values, needs, success, and rewards [23].

The effect of music on mood and emotion is a key aspect to consider when examining its use in the workplace. While there is limited research on the effects of music on satisfaction, it is reasonable to assume that listening to music can increase workplace satisfaction either directly or through other positive effects, most notably its ability to influence people’s emotions and overall well-being. This has been shown in other contexts: a recent study using an intelligent music-recommendation system to provide gym-goers with background music tailored to their physical exertion found that users of the system enjoyed their activities more [24]. Although job satisfaction does not end with mere enjoyment of the activity, the study reinforces the notion that there may be more effective types of music depending on arousal levels, even though the study only examined physical exertion during a particular gym activity and did not consider the emotional aspects of the participants.

Oldham and colleagues [25] found that music can influence the workers’ moods, which in turn affects their work outcomes. It should be noted that, in this work, both the experimental and control groups consisted of individuals who chose to listen to music at work. Other research has indicated that workers usually prefer to be able to listen to music because it makes work more enjoyable and increases satisfaction and creativity [21,22]. Another work has indicated that music at work evokes positive emotions as well as inspiration, concentration, and stress reduction [13]. Recent studies have indicated that listening to music can influence well-being through mood regulation [26] and its promoting effect on socialization [27]. In another study, short, live concerts at work were found to increase worker satisfaction by providing a moment of respite and/or socialization and strengthening the sense of belonging during the initial period of COVID-19 pandemic [28]. The ability to listen to preferred music at work also implies a degree of freedom and autonomy, which could further enhance individual well-being. Listening to music may also serve as a strategy for cognitive control of auditory space [29], which can be used to block unwanted distractions or, more generally, replace unpleasant activations with desirable ones.

### 1.3. The Use of Music

The work of Chamorro-Premuzic and Furnham [14] defines three different uses of music in everyday life. The first is emotional use, which can evoke positive or negative moods, change the emotional state itself, or evoke pleasure in experiencing an emotion that is not necessarily positive. The second is cognitive/rational use, which is the extent to which people listen to music for intellectual purposes and enjoy the more technical aspects; this may be related to the aesthetic perception described earlier. The final category is background use, which refers to the extent to which people listen to and enjoy music while performing other tasks without being distracted. Although the questionnaire is not specifically tailored to the work context, the respective uses of music have been shown to correlate with individual characteristics such as intelligence, personality traits, and emotional intelligence [14,30].

As Halliday [31] noted, the everyday use of music can be seen as an indirect indicator of individual differences, which are most important when studying organisational outcomes. In their study, the authors found that the three types of music use were associated with a variety of organisational outcomes including satisfaction, work engagement, and innovation. Although the authors proposed a final model in which the three uses are combined into one variable, it can be argued that they should have different effects on different outcomes because they relate to different underlying processes.

In particular, emotional use should be consistent with the positive effects of music on mood. It should be noted that this use refers to the process of achieving a particular emotion, whether positive or negative. As Lesiuk [21] notes, even sadness can produce satisfaction when it is the target of emotional regulation while, more generally, mild positive activation can improve cognition and thus performance. We therefore argue that positive activation stemming from the emotional use of music will lead to increased job satisfaction. Using music as a means of emotional regulation may also lead to a sense of having more resources at one’s disposal, which may prove effective in enhancing perceptions of performance in two different contexts: during breaks, which can provide a moment to recover and manage one’s emotions, so as to return to work refreshed; and during performance itself, buffering boredom or other negative activation caused by work tasks. Furthermore, we argue that the emotional use of music also has an indirect effect on performance by increasing satisfaction, as job satisfaction has been shown to be positively related to performance [32,33,34].

**H1a.**  *Emotional use of music has a positive relationship to job satisfaction.*

**H1b.**  *Emotional use of music has a positive relationship to performance.*

**H1c.**  *Emotional use of music has an indirect, positive relationship to performance through the mediation of job satisfaction.*

As for the cognitive use of music, we expect it to have a positive effect on satisfaction as well; the opportunity to listen to intellectually fulfilling music should also be considered a positive activation, consistent with aesthetic perception [9], leading to a sense of satisfaction. On the other hand, we hypothesize that cognitive engagement is unrelated to performance. On one hand, focusing on musical structure and composition could reduce cognitive resources dedicated to work tasks. On the other hand, increased activation has the effect of limiting attention, which could have a positive effect on performance, depending on the task. However, we argue that the general population is less familiar with musical subtleties, and we expect this use to be less frequent and more likely to occur during moments of pause and social interactions than directly during work tasks. Instead, we propose that cognitive use has an indirect effect on performance by increasing job satisfaction.

**H2a.**  *Cognitive use of music has a positive relationship to job satisfaction.*

**H2b.**  *Cognitive use of music has no direct relationship to performance.*

**H2c.**  *Cognitive use of music has an indirect, positive relationship to performance through the mediation of job satisfaction.*

Finally, concerning background use of music, we hypothesize that there is no relationship with satisfaction or performance. Music as mere background music should not be sufficient to cause significant activation, since background use implies low distractibility. Therefore, we do not expect it to have a significant effect on organisational variables.

**H3a.**  *Background use of music has no direct relationship to job satisfaction.*

**H3b.**  *Background use of music has no direct relationship to performance.*

**H3c.**  *Background use of music has no indirect relationship to performance through the mediation of job satisfaction.*

## 2. Materials and Methods

### 2.1. Procedure

This exploratory work aimed to investigate the effects of three different music uses, as defined by Chamorro-Premuzic and Furnham [14], on the perception of job satisfaction and performance.

Data collection took place between March and May 2022 via an online questionnaire hosted on Google Forms. The research invitation was disseminated on social networks such as LinkedIn, and it was addressed to the general population of workers, whether or not they listened to music during their jobs. Professional musicians and, more generally, people for whom music is a job content were excluded from the sampling procedure. The cover letter emphasized the anonymous and voluntary nature of the participation and provided instructions for filling the questionnaire. We collected informed consent from every participant, including consent for publishing the aggregated data. Participants could opt out of the research without repercussions. Following data collection, the preliminary results were shared on LinkedIn as a moment of restitution for participants. The research was carried out in accordance with the Declaration of Helsinki [35], Italian regulations on data protection and privacy (Law 196/2003), and the GDPR [36].

### 2.2. Participants

A total of 444 workers participated in the research. We eliminated 20 cases, of which two did not consent to participate, seven did not consent to the use of their data for research purposes, and eleven did not consent to the use of their data in publications. Therefore, a total of 424 participants completed the questionnaire. Slightly more than half of the sample (57.7%) reported listening to music while working, 26.5% reported not listening to music because they could not, and the remaining participants (15.8%) stated that they did not want to listen to music. For the purposes of this study, only the 244 participants who listen to music while working are considered.

The sample was fairly balanced in terms of gender, with a slight prevalence of women (50.6%). The age of the respondents ranged from 18 to 67 years, with a mean age of 36.13 (SD = 11.91). More than half of the respondents were employed in the service sector, with 34.6% dealing directly with public clients and 26.4% in offices. The following more common sectors were industry or construction (18.6%), education and training (8.2%), health care (6.9%), and arts and entertainment (5.2%). The majority worked in the private sector (80.8%) with a permanent contract (47.7%), although 22.1% reported being freelance. Most were salaried employees (47.9%) with a full-time arrangement (75.7%). Participants worked an average of 4.06 days per week in attendance (SD = 1.97) and 1.45 days remotely (SD = 1.99). The average length of service was 9.52 years (SD = 10.34). Table 1 details the demographic and occupational characteristics of the sample.

Music was mainly listened to with headphones (37.9%) or played in an open-space office (34.2%).

### 2.3. Data Analysis

We conducted descriptive and correlational analyses using IBM SPSS, version 27 [37]. We then tested a full structural equation model adopting MPLUS 8 [38].

### 2.4. Measures

Job satisfaction was measured using five items from the job satisfaction scale of the COPSOQ II [39]. Respondents rated their level of satisfaction regarding various aspects of one’s own job, such as relationships, physical conditions, and prospects, on a Likert scale from 1 (“Very unsatisfied”) to 5 (“Very satisfied”). Cronbach’s alpha was 0.87.

Job performance was assessed with four items [40]. Respondents were asked to rate how effective they felt lately in some aspects of their job performance on a 5-point Likert scale, ranging from 1 (“Not at all”) to 5 (“Very much”). An example question was: “How effective were you in performing without mistakes?” Cronbach’s alpha was 0.88.

Music uses were constructed using fifteen items from Chamorro-Premuzic and Furnham [14]. Participants rated their agreement on how they use music on a 5-point Likert scale, from 1 (“Strongly disagree”) to 5 (“Strongly agree”). The scale had three underlying factors: the emotional use of music (items 1 to 5), rational/cognitive use of music (items 6 to 10) and background use of music (items 11 to 15). We assessed the reliability of each factor; one item from emotional use and one from background use were eliminated to increase reliability. The final Cronbach’s alphas were 0.78 for emotional use, 0.74 for cognitive use, and 0.72 for background use.

## 3. Results

Table 2 reports the means, standard deviations, and correlations of the study variables. Concerning the three uses, emotional use has the highest mean score, followed closely by background use, and cognitive use is decidedly less frequent; all uses are positively and significantly correlated with each other. Age is negatively correlated with all variables except cognitive use and performance, while being a man has a positive correlation with cognitive use and a negative with background use. Satisfaction and performance are positively correlated with both emotional and cognitive use of music, but not with background use.

A full structural equation model was tested with the three uses of music as independent variables; following the study by Chamorro-Premuzic and colleagues [30], we let the three uses correlate with age and gender as controls instead of exogenous variables. The model fitted to the data well (Table 3) with maximum-likelihood method (ML). The following goodness-of-fit criteria were considered: the χ^2^ goodness-of-fit statistic; the Root Mean Square Error of Approximation (RMSEA); the Comparative Fit Index (CFI); the Tucker–Lewis Index (TLI); and the Standardized Root Mean Square Residual (SRMR). Values >0.90/0.95 for the CFI and TLI indicate a good fit of the model, while values <0.05/0.08 of the RMSEA indicate an acceptable fit and <0.05/0.08 of the SRMR [41].

**Figure 1:**
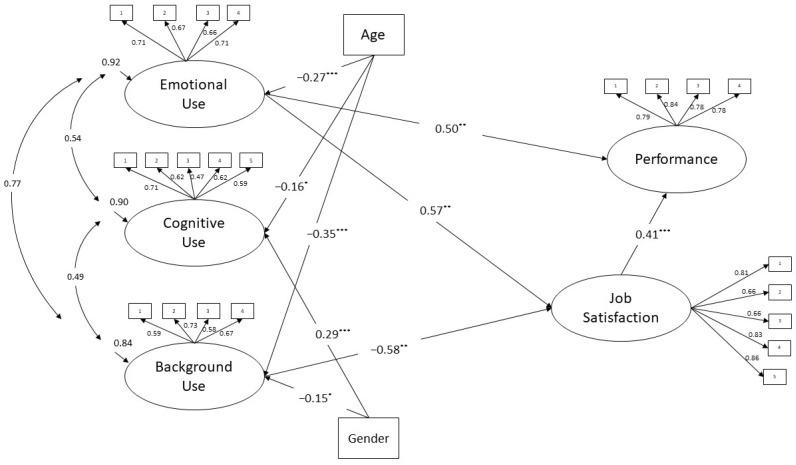
Model (ML estimation; standardized path coefficients; N = 235) controlling for age and gender (1 = Male). Non-significant relationships were not reported in the figure. * *p* < 0.05; ** *p* < 0.01. *** *p* < 0.001 for all factor loadings.

The full model with standardized path coefficient is reported in Figure 1. First, results showed that emotional use was directly positively related to both job satisfaction and performance, with which it also had a positive indirect relationship. Hypotheses 1a, 1b, and 1c were confirmed. Moreover, job satisfaction was positively related to performance. Second, cognitive use showed non-significant relationships to job satisfaction and performance, both directly and indirectly with the latter, thus disconfirming hypotheses 2a and 2c and confirming hypothesis 2b. Lastly, background use demonstrated a direct negative relationship to job satisfaction (disconfirming hypothesis 3a). The direct relationship to performance was not significant (confirming hypothesis 3b), while the indirect one was negative (disconfirming hypothesis 3c). Regarding the controls, age had a significant and negative relationship to all three uses, while gender had a negative relationship only to background use and a positive relationship to cognitive use.

Table 4 summarizes the indirect effects.

## 4. Discussion

This study explores the issue of listening to music while working, considering its uses and perceived effects, and provides a starting point for further investigation. Following the three uses of music described in the literature [14], it was interesting to observe how these different ways of listening to music may influence some important outcomes at work.

The emotional use of music seems to be the only one that has both direct and indirect positive effects on job satisfaction and performance. These results suggest that enjoying the emotions evoked by music might enhance the perception of satisfaction with one’s work, which also fosters a better perception of accomplishment of tasks required at work. Cognitive use, which was more common among men than women in our sample, is surprisingly not significantly related to job satisfaction. This could be related to the low scores for this type of music use in our sample. In addition, this paper intentionally excluded professional musicians, a group that values this type of music use more and therefore engages with it more frequently.

Because the cognitive use of music could be considered an additional requirement at work, listening to music that focuses on its structural aspects could distract from the pursuit of more important work-related tasks and counteract the possible positive activation from appreciating the structure rather than the content of the music. Another possible explanation is that the pleasant activation of cognitive use of music, which evokes a positive emotion, might be confounded with emotional use, which is far more common in this sample. Or, to put it another way, while cognitive use may imply a positive arousal that is inextricably linked to understanding the complexity of the music, the opposite cannot be said for emotional use. Finally, regarding background use, it seems to have only negative effects in our sample, both directly on job satisfaction and indirectly on performance. Given the high correlations with emotional use, it seems that background use in this sample involves some aspect of emotional regulation in music listening. Furthermore, because this study cannot imply causal relationships due to its cross-sectional design, it could be that the more dissatisfied people are with their work, the more they use music as a background; the same could be the case when performance decreases, which in turn leads to less satisfaction.

In terms of age, it appears that older people report lower use of music across the three uses. A relationship already found for background use that can be explained by the fact that younger people may be more responsive and have higher music consumption, which incidentally is also related to the three uses of music and age [30]. Finally, men seem to be more engaged in the cognitive use of music than women, whereas the opposite is true for background use.

### 4.1. Limitations

This study is not exempt from some limitations. First, due to the cross-sectional design, it is not possible to define causal effects between variables [42]; it will be necessary to proceed with longitudinal studies to overcome this limitation. Second, although the sample consisted of individuals who listen to music at work, unlike that of Chamorro-Premuzic and Furnham [14], it appeared to be inhomogeneous, especially regarding age and type of job. In addition, other important variables involved in workplace dynamics (e.g., resources or demands) might better explain the role of workplace music-listening in workers’ well-being.

### 4.2. Future Research

Based on these initial findings, future research could further investigate the role of music-listening in the workplace. Following the Job Demand–Resources theory [43], music listening could be perceived as a resource in the workplace, as our results suggest that the emotional nature of enjoying music improves job satisfaction and performance. However, this role could be more nuanced when considering these relationships in different work contexts (e.g., service sector or factory) and with different levels of autonomy. Future research should also explore the possibility of a bidirectional relationship between music use and organisational outcomes.

## 5. Conclusions

The role of music listening in today’s workplace is still unclear. Given the ease of access to multimedia content, the ability to isolate from outside noise and chatter, and the opportunity to enjoy music, especially in environments with high work autonomy, understanding the benefits of listening to music in the workplace could improve the work experience of workers, especially those who use music for emotional regulation. This understanding could also contribute to a shift in thinking such that music is not only used as a one-time intervention to calm anxiety [44], but could be a work practice that is more widely accepted and shared by management.

From a management perspective, it would be interesting to examine employee preferences for listening to music in the workplace, both in terms of listening modality and genre, and their impact on organisational outcomes. Indeed, meeting music-listening preferences could be another environmental characteristic of some workplaces that enhances employee autonomy, which, according to self-determination theory [45], could be a motivational boost and thus improve both well-being (i.e., job satisfaction) and job performance. The results suggest that simply listening to music in the background does not harm productivity per se, but it may not have a positive effect on job satisfaction, which is an important variable for performance. Rather than mandating listening to music in the workplace, management should work with employees to implement personalized interventions.

Lastly, considering situations in which listening to music at work is not possible, it would be noteworthy to deepen the reasons for why this is the case. Sometimes it is obviously related to the nature of the job itself, which may not allow one to isolate oneself and listen to music (e.g., in customer service), but in many occupations and workplaces where the work characteristics would allow this practice it is nevertheless not always performed. Thus, it may be that there are some obstacles or resistance from supervisors that cement the view that listening to music at work is counterproductive.

However, if further in-depth studies tailored to the work context confirm some of the findings, listening to music could be considered a “small” (but smart) support in building a personalized and enjoyable work situation for people at work. Therefore, it is crucial for research in this area to achieve appropriate dissemination with special reference to employers and HR functions.

## Figures and Tables

**Table 1:** Demographic and professional characteristics of the sample.

Variable	Categories	Percentage/Mean
Professional sector	Industry/Construction	18.6%
	Services (customer interaction)	34.6%
	Services (office)	26.4%
	Education and training	8.2%
	Arts and entertainment	5.2%
	Health care	6.9%
Type of sector	Public	19.2%
	Private	80.8%
Type of contract	Permanent contract	47.7%
	Temporary contract	17.0%
	Apprenticeship/Training	10.6%
	Freelancing	22.1%
	Other type of contract	2.6%
Professional status	Executive	4.2%
	Upper manager	3.3%
	Middle manager	4.2%
	White collar worker	47.9%
	Blue collar worker	12.9%
	Not applicable	27.5%
Work regime	Full-time	75.7%
	Part-time	13.8%
	Not applicable	10.5%
Seniority		M = 9.52; DS = 10.34
Weekly workdays	In presence	M = 4.06; DS = 1.97
	Remote	M = 1.45; DS = 1.99

**Table 2:** Means, standard deviations, correlation, and reliability coefficients.

	M	DS	1	2	3	4	5	6	7
1. Age	36.13	11.9	1						
2. Gender (1 = Men)			0.12	1					
3. Emotional use	3.58	0.91	−0.23 **	−0.09	***0.72***				
4. Cognitive use	2.47	0.89	−0.07	0.25 **	0.38 **	***0.74***			
5. Background use	3.34	0.91	−0.30 **	−0.17 *	0.60 **	0.32 **	***0.72***		
6. Satisfaction	3.43	0.96	−0.13 *	0.01	0.26 **	0.18 **	0.03	***0.87***	
7. Performance	3.79	0.84	−0.07	−0.01	0.31 **	0.14 *	0.10	0.51 **	***0.88***

* *p* < 0.05, ** *p* < 0.01. Reliability coefficients are reported in the diagonal in bold italic.

**Table 3:** Fit indices of the three models tested.

χ^2^	df	*p*	CFI	TLI	RMSEA	SRMR
429.106	233	<0.001	0.90	0.89	0.06 (CI: 0.05, 0.07)	0.07

**Table 4:** Indirect effects of the model.

Indirect Effect	Est.	S.E.	*p*
Emotional Use -> Job Satisfaction -> Performance	0.23	0.08	<0.001
Cognitive Use -> Job Satisfaction -> Performance	0.08	0.05	ns
Background Use -> Job Satisfaction -> Performance	−0.24	0.08	<0.01

## Data Availability

The data presented in this study are not publicly available due to the Italian privacy law. The data are available on request from the corresponding author.
